# ASSIST: a reablement program for older adults in Sweden – a feasibility study 

**DOI:** 10.1186/s12877-022-03185-2

**Published:** 2022-07-26

**Authors:** Susanne Assander, Aileen Bergström, Christina Eriksson, Sebastiaan Meijer, Susanne Guidetti

**Affiliations:** 1 Department of Neurobiology, Caring Sciences and Society, Division of Occupational Therapy, Karolinska Institutet, Stockholm, Sweden; 2 Academic Primary Health Care Centre, Stockholm, Sweden; 3 Department of Biomedical Engineering and Health Systems, Kungliga Tekniska Högskolan, Stockholm, Sweden; 4 Medical Unit Occupational Therapy and Physiotherapy, Theme Women´S Health and Allied Health Professionals, Karolinska University Hospital, Stockholm, Sweden

**Keywords:** ADL, Aging in place, Home rehabilitation, Home care services, Information- and communication technology, Occupational therapy, Person-centred care, Quick response code, Restorative care

## Abstract

**Background:**

Western countries emphasise the provision of assistive home care by implementing reablement services. Reablement services are offered to a limited degree in Sweden, and systematic research regarding outcomes and how reablement can be tailored to maximize benefits for older adults has been lacking. This study aimed to evaluate the feasibility of a novel reablement program (ASSIST 1.0) regarding study design and outcome measures, as well as fidelity, adherence, and acceptability of the program in a Swedish context.

**Method:**

A non-randomised, quasi-experimental, mixed-method, pre/post-test design was applied with an intervention group receiving ASSIST 1.0 (*n* = 7) and a control group receiving regular home care (*n* = 10). ASSIST 1.0 was developed to empower older adults to increase their perceived performance and satisfaction of performing activities in everyday life as well as increase their perceived health, self-efficacy, and well-being. ASSIST 1.0 was founded on the concept of reablement and included three components: i) goal setting with The Canadian Occupational Performance Measure (COPM), ii) provided support to home care staff to enhance their provision of reablement, and iii) explored the incorporation and use of an information- and communication technology (ICT) to facilitate information transfer.

**Results:**

Using COPM for goal setting with older adults and providing support to the staff via workshops were valuable components in the delivery of ASSIST 1.0. The ICT product encountered several challenges and could not be evaluated. COPM and EQ-5D were deemed the most important instruments. Organisational and political barriers affected the feasibility. Although, the fidelity and adherence were complied the staff perceived the program to be acceptable.

**Conclusion:**

The ASSIST 1.0 program was feasible in regard of study design, delivering the intervention, and evaluating instruments that detected a change. A logical progression would be to conduct a full-scale trial. In addition, a usability study to evaluate the technological component is also recommended. With minor improvements, the ASSIST 1.0 program has the potential to contribute to the development of a home care organisation that could enhance older adults possibility to age in place at home.

**Trial registration number:**

NCT03505619

## Background

The risk of having issues that negatively influence one´s ability to perform activities increases with age; with consequences for hospitalizations or moving to nursing homes [1]. A reduced ability to perform activities in everyday life often leads to a need to receive home care services. However, to provide necessary support and facilitate ageing in place for older adults so they can live and thrive at home, a structured and well-organised social and health care system is required [2, 3].

In Sweden, home care services generally provides a passive support, where home care staff do activities for the older adults rather than providing an assisting support with focus on the older adults´ potential. The World Health Organization (WHO) and the European Commission (EC) emphasizes older adults´ potential for physical, social, and mental well-being, suggesting that older adults should be enabled to participate in activities in everyday life both at home and in society [4, 5]. To enable participation in activities, the EC recommends that health and social care services provides reablement [4].

Reablement (or restorative care) has internationally been defined as a person-centred and holistic approach that aims to maintain or increase older adults´ independence in meaningful activities in everyday life and reduce their need for long-time services. The service is provided during multiple visits and founded on a goal-oriented plan [6]. The approach facilitates an active involvement in and performance of activities in everyday living, as well as participation in society [7]. Research has shown that older adults who received reablement increased their perceived life quality [1, 8–10], mental and physical health, and independence to conduct everyday activities [11, 12] compared to those who received regular home care services. Utilizing a reablement approach also showed that the probability of older adults being admitted to hospital or nursing home [12–14] and in need of home care [15] decreased, while their possibility to live at home increased [11]. Reablement could therefore be considered an approach that would support the development of a structured and sustainable health and social care services, and thereby increase the opportunity for older adults to age in place.

Several western countries have implemented reablement to emphasise assistive support to reduce older adults´ dependency on care [1, 12, 13, 16–20]. The reablement programs have differed to some extent between and within countries [21], e.g. some of them have included a multidisciplinary or interdisciplinary team [22–25], while others have been provided by nurse assistants in home care settings [14, 20, 26]. Programs has to be tailored to the specific context [27] and take existing health care system, legislation, and resources into account [21]. Although, there is a lack of conformity regarding the outcomes of reablement because the effectiveness and outcomes vary in different contexts [7–14, 27]. Therefore, more research is needed on how the benefits of reablement can be maximized in different contexts [15, 29, 30].

In Sweden, care for older adults is financed by general taxes and older adults have the right to care if a formal assessment concludes such a need. However, local authorities determine what is considered a need and how this need is to be met [31]. Furthermore, in recent decades there have been organisational and regulatory changes within the home care system, which has led to an increased strain on maintaining a sustainable service [32]. Today, the Swedish home care service is comprehensive and includes housekeeping services, assistance in activities of daily living (ADL), and health and social care service [33, 34]. In addition, there is a divergence between the number of older adults in need of the service and their preferences for the received service, and staffing resources and time allocated for the staffs visits to the older adult [35].

Until today, reablement have only been tested and applied to a limited extent in some Swedish regions [22, 36]. Although the approach has been discussed for more than a decade within the Swedish health and social care sector [37, 38] no agreement has been reached on what reablement should contain [22, 36]. In addition, more research is needed on how reablement can be delivered by home care staff [15, 29, 30]. In response to the lack of systematic research and the limited knowledge and use of reablement in Sweden, a novel reablement program ASSIST 1.0 was developed [7]. ASSIST 1.0 was founded on the concept of reablement (presented earlier) and underpinning theories of The Canadian Model of Occupational Performance and Engagement and the ‘Do, Live, Well’ framework [7]. Except from providing a reablement approach, ASSIST 1.0 constituted of the components: setting goals and designing the intend support with the older adult, supporting the staff who were providing the intervention to the older adults, and incorporation of information- and communication technology (ICT) to enhance the flow of information for those providing support to the older adult. The hypothesise was that the use of ASSIST 1.0 would reinforce older adults potential to regain, maintain, or improve their skills to perform and participate in everyday activities as well as increase their perceived health, self-efficacy, and well-being.

ASSIST 1.0 was a new multidimensional program assessed in a complex context. ASSIST 1.0 was therefore initially conducted as a feasibility study, as recommended by the Medical Research Council (MRC) guidelines [39]. A feasibility study enables the possibility to identify barriers and facilitators to improve the program before a full-scale trial is conducted.

### Aim

This study aimed to evaluate the feasibility of a novel reablement program, ASSIST 1.0, for older adults in a Swedish context in terms of study design and outcome measures, as well as the program´s fidelity, adherence, and acceptability.

## Method

### Study design

This was a non-randomised, quasi-experimental, mixed-method study with a pre/post-test design which involved one intervention group (IG) who received ASSIST 1.0 and one control group (CG) who received regular home care services in the region of Stockholm, Sweden. The study followed the CONSORT 2010 statement for randomised pilot and feasibility studies [40] and was registered as a clinical trial (NCT03505619) 23/04/2018. Ethical approval was obtained from the Swedish Ethical Review Agency (Registration nr: 2017/1439–31/1 and nr: 2018/2691–32).

### Context of the study

Two geographical areas in the Stockholm region were selected via convenience sampling. The area that represented the IG had a supportive discharge home care service (SDHCS) group, which consisted of four nurse assistants. SDHCS groups have been implemented in some Swedish municipalities. The intention with establishing SDHCSs have been that older adults should feel safe and secure when they return home from inpatient care, reduce their dependence on care, and increase their participation and independence. To enable this, SDHCS staff have had a rehabilitative approach and supported older adults to maintain their abilities, function, and independence. For a period of two weeks, the SDHCS staff provided extensive and intensive support and coordinated various medical contacts when needed. For this study, an SDHCS group was enrolled as gatekeepers (mediators to access potential participants), as well as providers of ASSIST 1.0 to the older adults who were included in the IG.

In the CG, an administration team was enrolled as gatekeepers. They assessed and granted home care services to older adults when they were about to be discharged from the hospital. This geographical area had not implemented SDHCS, hence, the included older adults could only receive regular home care.

When the gatekeepers first encountered the older adults at the hospital, they gave a brief oral introduction and an information booklet about the project. If the older adult wanted to participate, they could contact the researcher themselves or get support from the gatekeepers to get in touch with the researcher before discharge. Then, when the older adult was discharged, the researcher (first or third author) met them in their homes where they received written and oral information about the study as well as information about their possibility to withdraw without this affecting their provision of SDHCS or regular home care. If they then met the eligibility criteria, they were asked to sign a consent form, after which the first assessment began.

### Eligibility criteria

**Fig. 1:**
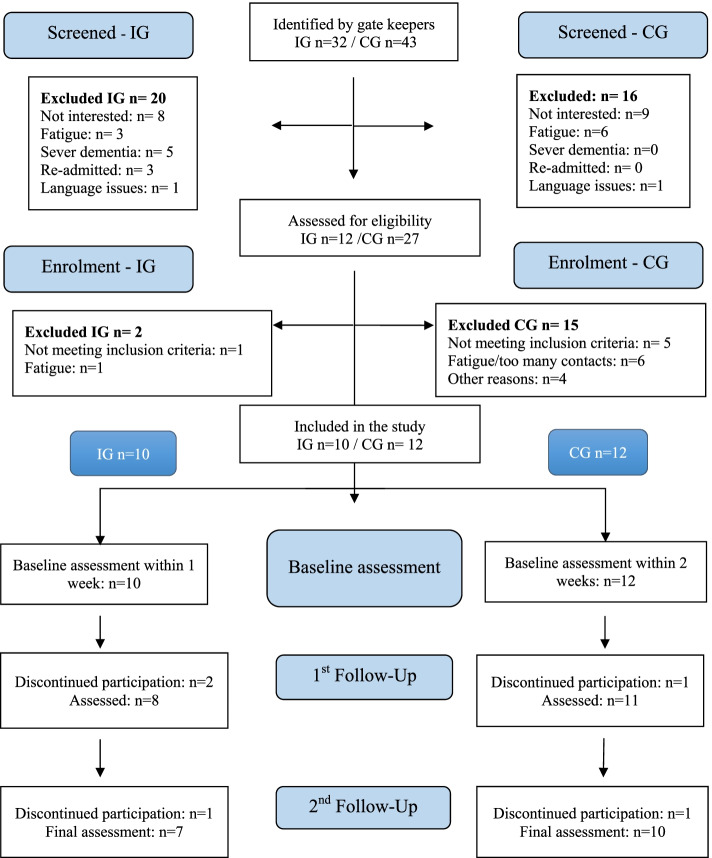
CONSORT Flow Chart – recruitment process for IG and CG

Inclusion criteria for all the older adults were: 1) being over 65 years of age, 2) discharged from the hospital, 3) granted home care service, 4) spoke and understood Swedish, and 5) could conduct the first assessment within five days of discharge from the hospital. Participants in the IG also had to be granted SDHCS after discharge. The exclusion criteria were: 1) having cognitive dysfunctions which hindered the person from understanding their situation, answering questions, or not being able to formulate goals, and 2) receiving home health care (Fig. 1).

### Amended eligibility criteria

After the inclusion process had started, two eligibility criteria were amended. It was considered that the reception of home health care did not interfere with the program, therefore it was removed as an exclusion criterion. For the CG, the inclusion criterion regarding the time for the first assessment was adjusted from five days to ten days because they did not receive initial specific services, such as the SDHCS, at discharge.

### The ASSIST 1.0 program

The ASSIST 1.0 program has been presented elsewhere [7], where the original idea was to have regular home care staff providing the intervention to the older adults. However, in this feasibility study, ASSIST 1.0 was provided by an SDHCS group (introduced above) which required that the intervention process was remodelled.

#### Context and reablement

Prior the start of the intervention, the first and third author presented the content of ASSIST 1.0 and the concept of reablement to the SDHCS group.

When providing reablement, the staff was instructed to apply a proactive and preventative approach where they should support the older adult to live as independent as possible. The staff encouraged the older adult to perform everyday activities with them, or on their own, instead of the staff performing activities for or to the older adult [7, 30, 41]. Besides working in accordance with reablement, the ASSIST 1.0 program included three additional components: i) to formulate goals for activities of everyday living together with the older adult using the Canadian Occupational Performance Measure (COPM) [42, 43]; ii) to provide support to the SDHCS group to reinforce their provision of reablement to older adults; iii) an ICT product i.e. Quick Response (QR) codes that would enable information sharing about set goals and needed support.

Reablement is usually provided for a period of 8 – 10 weeks, although, in this study the SDHCS group only provided their services for two weeks. Hence, the information about set goals, requested support and how to empower the older adult in activities in everyday living was planned to be passed on to the regular home care team that took over the support. This was conducted orally or in writing at a handover meeting. In addition, written information was provided about the QR-codes and how they would be used. The older adult would then continue to work with the set goals together with the regular home care staff until the final follow-up, 8 weeks later. The regular home care teams that took over, did not receive any support from the researchers.

#### Goal setting in activities of everyday living

During the baseline assessment, the researchers used COPM to identify areas in everyday living where the older adult perceived performance problems in the areas of self-care, productivity, or leisure [42, 43]. The identified activity problems were transformed into goals that aimed to regain, maintain, or improve the older adult´s ability to perform the chosen activities. Strategies for how the older adult would achieve these goals were formulated in collaboration between the assessing researcher and the older adult. The goals and strategies were then shared with the SDHCS group so that they could provide a person-centred and tailored support according to the needs and wishes of the older adult.

#### Reinforced support to the SDHCS

Throughout the intervention period, workshops for the SDHCS group would be conducted once a week with an occupational therapist (OT) (third author) [7]. The content of these sessions was contemplated to be developed in collaboration with the staff and was intended to support the utilization of reablement and tailor the intended support to the older adults within the ASSIST 1.0 program. In addition, the SDHCS would receive ‘hands-on’ coaching in the home of the participating older adults by the OT. These two approaches were included since it has been proposed that professional coaching significantly can increase awareness of one´s actions at work [7, 44].

#### QR-codes

To facilitate the transfer of information on set goals and needed support between SDHCS, regular home care service, and informal carer providers, an ICT product consisting of a QR-code was included in ASSIST 1.0. QR-codes are a type of barcode that consists of encoded data that can be scanned by, e.g., a smartphone, and links to a website where information is accessed [45]. The hypothesis was that QR-codes would facilitate easy and quick access about goals and needed support that each older adult had decided on, and would also reduce recurring questions by staff who visited the older adult. The QR-codes would also enable the older adult to share the information with whomever they wanted. In addition, since most older adults did not have a smartphone, the researchers developed a document with pictures and text as a visual reminder for the older adult with the information that had been incorporated into the QR-code.

### The control group

Older adults in the CG only received regular home care service after discharge.

### Data collection

All data collection with the older adults occurred face-to-face in their home and was conducted by the first author with the CG and the third author with the IG. Basic demographic data were collected during the baseline assessment. Quantitative data focusing on the older adult’s perception of their situation and perceived ability to perform activities in everyday life was collected at the baseline and follow-up session ten weeks later. During the follow-up session, qualitative interviews were also conducted to capture the older adult´s experience of returning home and their reflections on the support they had received.

Interviews with each nurse assistant in the SDHCS group were conducted in their office by the first author before the ASSIST 1.0 program started, six months later, and approximately one year after the start of the study program. An interview with the manager of the SDHCS was conducted by the second author when ASSIST 1.0 had ended.

In addition, the first and the third author conducted participant observations and wrote logbooks from all meetings, as well as after being in contact with the older adults, the SDHCS group and their manager, or with the administration team.

To evaluate the use of the QR-codes, data was collected from the web platform where the information to the QR-codes was created. This data consisted of login frequency and who had visited the webpage. In addition, questions regarding the use of QR-codes were included in the interview guides for the nurse assistants in the SDHCS group and for the older adults in the IG.

### Feasibility of ASSIST 1.0

#### Study design

The study design was assessed in terms of recruitment and retention, conducted activities and contextual aspects [46] to provide insights of facilitators and barriers of the intervention. Activities conducted within the program were assessed with data from logbooks and analyses of the interviews with the staff in the SDHCS group. Logbooks and interview data were also used to capture contextual aspects such as organisational, ethical and political [46, 47] that could have affected the implementation of ASSIST 1.0. Data from the QR-codes were analysed to evaluate how frequent the QR-codes were used and by whom.

#### Clinical outcome measures

The instruments were evaluated by assessing their capability to detect change after ten weeks. Initially, ten instruments were included to evaluate various aspects of older adults´ health, well-being, activity performance, self-efficacy, and quality of life. These instruments were: COPM measuring the perceived performance and satisfaction of performing chosen everyday activities [43], Barthel/Katz Extended Activities of Daily Living (ADL) index measuring (in)dependence in ADL [48, 49], Frenchay Activity Index (FAI) measuring participation of performing activities in everyday life such as domestic chores, social and leisure activities, and community participation [50], a Self-Efficacy Scale (SES) measuring the perceived belief in one’s ability in different everyday activities based on Banduras theory [51], Reintegration to Normal Living (RNL) measuring community integration [52], Hospital Anxiety and Depression Scale (HADS) measuring anxiety and depression [53], Life Satisfaction Questionnaire (LiSat -11) where only the global satisfaction with life item was used [54], EQ-5D-3L and EQ-VAS measuring self-reported health-related quality of life [55] and Sense of Coherence (SOC-13) measuring one’s sense of health [56–58].

After enrolment of the first two participants in the IG, two instruments were adjusted and two instruments were added to the study. The adjustment consisted of asking the participants to rate their (in)dependence five days before admission to the hospital with the Barthel Index and Katz Extended ADL index. The additional instruments were the World Health Organization Disability Assessment Schedule 2.0 (WHODAS 2.0) a generic assessment instrument that measures health and disability [59] and the Darthmouth Functional Health Assessment Chart/WONKA (COOP/WONKA) measuring functional health and well-being [60]. Both instruments have been used in previous reablement studies [13, 61–63].

All instruments, the scoring, and when they were used are presented in Table 1.

**Table 1:** Instruments used for data collection, purpose, measurement aspect, cut-of score/clinically significant value and sequence of assessment

Assessment sequence	Instrument	Purpose	Measure	Cut-of/clinical significance	Feasibility evaluation	Baseline	1st follow-up	2nd follow-up
Baseline 1st follow-up 2nd follow-up	Canadian Occupational Performance Measure (COPM)[42]	Perceived performance and satisfaction with activities related to self-care, productivity, and leisure	Scale ranging from 1 to 10 in two aspects, i) current performance, 1 = not able to perform the activity at all to 10 = able to do it extremely well, and ii) satisfaction with doing, 1 = not satisfied to 10 = extremely satisfied	A 2-point change between measure points = clinically significant change	Feasibility of the outcome measure	X	x	x
Baseline 2nd follow-up	Barthel Index [48]	The psychical function of personal activities in daily life and instrumental activities in daily life	0 = dependent, 5 = need of assistance and 10 = independent. Total sum ranging from 0–100		Feasibility of the outcome measure	X		X
Baseline 2nd follow up	KATZ – ADL [49]	The psychical function of personal activities in daily life and instrumental activities in daily life	0 = dependent or 1 = independent. Total sum ranging from 0–10		Feasibility of the outcome measure	X		X
Baseline 2nd follow-up	Frenchay Activity Index (FAI) [50]	Frequency of conducting a variety of social and domestic activities during the last 3 or 6 months	Scale ranging from 0 to 3 for each question. The total sum ranges from 0 = inactive to 45 = very active		Feasibility of the outcome measure	X		X
Baseline 2nd follow-up	Self-efficacy Scale (SES) [51]	Confidence in the ability to conduct activities	Each activity is rated: 1 = not confident to 10 = very confident Total sum ranging from 18–180	A score of > 5 is considered to represent confidence in the ability to perform activities in daily life	Feasibility of the outcome measure	X		X
Baseline 2nd follow-up	Re-integration to normal living index (RLNI) [52]	Mobility, self-care, daily activity, recreational activity, and family roles	Each question is rated: 1 = does not describe my situation to 4 = describes my situation very well Total sum ranging from 11–44		Feasibility of the outcome measure	X		X
Baseline 2nd follow-up	Hospital Anxiety and Depression Scale (HADS) [53]	Perceived anxiety and depression	Two subscales: anxiety and depression. Responses are graded 0 to 3. Subscale scores range from 0–21	0–7 = no anxiety and depression, 8–10 = mild, or 10–21 = moderate to severe anxiety and depression	Feasibility of the outcome measure	X		X
Baseline 2nd follow-up	Life Satisfaction Scale 11 (LiSat-11)	Perceived satisfaction with life	Scale 1–6: 1 = not satisfied to 6 = very satisfied		Feasibility of the outcome measure	X		X
Baseline 2nd follow-up	EQ-5D-3L [55]	General life quality. Perceived state of health in five aspects: mobility, hygiene, main activities, pain, and anxiety	Index scale from 0 to 1. 0 = death and 1 = full health	A change of more than 0.1 = clinically significant change	Feasibility of the outcome measure	X		X
Baseline 2nd follow-up	EQ-Visual Analogue Scale (EQ-VAS) [55]	Perceived state of health	Scale 0–100: 0 = worst possible health, 100 = best possible health	A change with more than 10 = clinically significant change	Feasibility of the outcome measure	X		X
Baseline 2nd follow-up	Sense of Coherence 13 (SOC- 13) [56, 57]	Perception of one´s existence/coherence in life	Each question is rated on a 7-point Likert scale. The total sum ranges from 13 to 91. A higher score = a greater sense of coherence	A mean of 61 is considered normal in Sweden	Feasibility of the outcome measure	X		X
1st follow-up 2nd follow-up	WHO Disability Assessment Schedule 2.0 (WHODAS 2.0) [59]	Identifies difficulties in daily life due to the state of health during the past 30 days	12 questions with responses on a 5-point scale ranging from 1 = no problem to 5 = extreme problem/cannot perform. Total sum ranging from 12–64		Feasibility of the outcome measure		X	X
1st follow-up 2nd follow-up	The Darthmouth Functional Health Assessment Chart/WONKA (COOP/WONKA chart) [60]	Perceived functional capacity during the last two weeks	5 questions responded on a 5-point scale ranging from 1 = no limitation/much better at all to 5 = severely limited/much worse. Total sum ranging from 5–25		Feasibility of the outcome measure		X	X
Before/during/ after the project.	Semi-structured interviews					Before projects start	After 6 months	After project ended
	- SDHCS	To explore the staffs' perception and experiences of the project.			Evaluate the project process and the fidelity, adherence, and acceptability of the intervention			
	- SCHCS Manager	To explore the managers' perception and experiences of the project.						After project ended
Throughout the project	Logbooks by							
	- Researchers/Occupational Therapist	Reflections and tracking of the project process				During the whole project		

#### Fidelity and adherence

Fidelity and adherence of the ASSIST 1.0 program was identified by analysing the interviews with the SDHCS group (which occurred during and after the intervention), the interviews with the older adults in the IG, as well as the content in the logbooks. The logbooks included information about e.g., planned and executed workshops and coaching sessions, attendance at and themes for the workshops, strategies of the provided support to the older adult, and the older adult’s engagement when working with their set goals.

#### Acceptability

Acceptability was determined through information retrieved from the interviews with the staff in the SDHCS group and their manager.

### Data analysis

Descriptive statistics were used to describe information regarding the recruitment, characteristics of the older adults, and scores from outcome measures. All outcome data from the instruments were normally distributed, hence, inferential statistics as independent and paired sample t-tests were applied. T-tests were applied to explore the sensitivity of the instruments regarding the differences of mean scoring between baseline and follow-up assessments within and between the IG and CG. Differences were significant at *p* < 0.05 and clinical significance was presented for instruments when applicable. IBM SPSS Statistics version 26 [64] was used to analyse the data.

Logbooks and interviews were analysed according to a content analysis conducted by the first author. The text in the logbooks and the content of the interviews was compared to the descriptions of fidelity, adherence, and acceptability, as well as in relation to the components of ASSIST 1.0.

## Results

### Study design

#### Recruitment and retention

Enrolment of older adults occurred during one year for each group, November 2018 – November 2019 (IG) and March 2019 to March 2020 (CG). The gatekeepers identified 75 potential participants, however, 36 were excluded upon discharge e.g., due too fatigue at discharge or not interested to participate immediately after discharge. Of the remaining 39 older adults (IG *n* = 12, CG *n* = 27), the researcher assessed their eligibility via a phone call or at the first home visit. Another 15 older adults were excluded from the CG because they did not meet the inclusion criteria e.g., had declined to receive home care, were judged to have too many contacts with healthcare professions after discharge, or felt too fatigued to participate. Finally, 22 older adults were enrolled: ten to the IG and twelve to the CG (Fig. 1). The total recruitment rate was 56% and the drop-out rate was 30% in the IG and 17% in the CG. Reasons for drop-outs were e.g., hospital readmissions or lost interest. Hence, seven participants completed their participation in the IG and ten in the CG.

#### Participants

A majority of the participants were women and the mean age was 87 in the IG and 86 in the CG. The participants in the IG had been hospitalized for a longer period (56% > 15 days) and perceived a poorer state of health after discharge, compared to those in the CG (Table 2).

**Table 2:** Demographic and characteristics of participants in the IG and CG at baseline

Variables	Intervention group *n* = 7 (%)	Control group *n* = 10 (%)
Gender		
* Female*	5 (70)	7 (70)
Age [range]	87 [78–94]	86 [70–92]
Housing		
* Apartment*	7 (100)	6 (60)
* House*	-	4 (40)
Civil status		
* Married*	-	3 (30)
* Living apart*	1 (14)	-
* Living alone/Widow-er*	6 (85)	7 (70)
Main occupation		
* Retired*	7 (100)	8 (80)
* Worker*	-	2 (20)
Received home care during the last year		
* Yes*	6 (84)	4 (40)
Hospital admissions during the last year		
* 1*	4 (56)	8 (80)
* 2*	3 (42)	2 (20)
* 3 or more*	-	-
Days spent at the hospital at recent admission		
* 1 – 7*	2 (28)	3 (30)
* 8—14*	1 (14)	5 (50)
* 15 – 28*	1 (14)	-
* Longer*	3 (42)	2 (20)
Amount of home care today ^a^		
* 1–3 times daily*	2 (28)	7 (70)
* 4–6 times daily*	4 (56)	3 (30)
Receives support from friends and/or family		
* Yes*	7 (100)	8 (80)
Perceived health		
* Very well*	*-*	*-*
* Well*	-	1 (10)
* Okay*	4 (56)	8 (80)
* Bad*	3 (42)	-
* Very bad*	-	1 (10)
Perceived health compared to others at the same age ^b^		
* Much better*	-	1 (10)
* A little better*	3 (42)	4 (40)
* The same*	1 (14)	1 (10)
* A little worse*	1 (14)	1 (10)
* Much worse*	2 (28)	1 (10)

Missing value: number of missing participants indicated with a = 1 person, b = 2 persons

^a^) One person in the IG could not estimate the amount

^b^) Two persons in the CG did not have anyone to compare with

#### Conducted activities and context

Data in logbooks and interviews showed that the workshops provided by the OT facilitated the SDHCS group to discuss and reflect on their working methods and the process of delivering ASSIST 1.0. The sessions were intended to be co-created, but the staff expressed that it was difficult to ask for topics they did not know anything about. Therefore, the researchers gave suggestions for topics to be discussed that were aligned to the content of ASSIST 1.0, such as working according to and applying a reablement approach, and how to formulate realistic and achievable activity goals with older adults.

The intended coaching sessions in the older adults home did not go as planned. Coaching sessions were conducted with four participants (57%), once with each. For the remaining three participants, problem arose in coordinating the time for these occasions.

In addition, after the first four months, organisational and political contextual aspects created barriers that interfered with the design and led to a diminution in having weekly workshops and including participants. These barriers consisted of reduced financing and reduced number of employees, as well as revision of the SDHCS duties.

Furthermore, the incorporation of QR-codes met several barriers and, in the end, it was not possible to evaluate the effect of including QR-codes. Despite this, the SDCHS group and their manager expressed enthusiastic comments in their interviews regarding the possibilities the QR-code could provide and believed that QR codes could be of value and facilitate their work in the future.

#### Clinical outcome measure

The instruments capability to detect changes between and within the groups after ten weeks was evaluated in relation to clinically and statistical significance within and between the groups (Tables 3, 4 and 5). The responses in WHODAS 2.0 and COOP/Wonka was given in regards of how the situation had been during the recent weeks; hence, the change was measured between the second and tenth week of the ASSIST 1.0 program.

**Table 3:** Mean difference between baseline and the ten-week follow-up assessment within each group

Measures	IG				CG			
	Mean (SD)	t	df	*P*-value	Mean (SD)	t	df	*P*-value
COPM Performance (1–10) ^↑^	3.37 (2.28)	3.74	6	0.01^*^	2.91 (3.73)^b^	2.21	7	0.06
COPM Satisfaction (1–10) ^↑^	4.40 (2.98)	4.18	6	0.01^*^	3.23 (2.93)^b^	3.12	7	0.02^*^
Barthel Index (0–100) ^↑^	27.85 (29.56)	2.49	6	0.05^*^	11.50 (7.47)	4.87	9	0.00^*^
KATZ (0–10) ^↑^	2.43 (2.76)	2.33	6	0.06	1.40 (1.84)	2.41	9	0.04^*^
FAI (0–45) ^↑^	-7.14 (8.40)	-2.25	6	0.07	-3.50 (5.48)	-2.02	9	0.07
SES (18–180) ^↑^	33.50 (38.65)^a^	2.12	5	0.09	30.10 (25.77)	-3.69	9	0.01^*^
RNLI (11–44) ^↑^	4.43 (4.27)	2.74	6	0.03^*^	1.30 (7.07)	0.58	9	0.58
HADS A (0–21) ^↓^	0.00 (2.52)	0.00	6	1.00	-1.60 (1.51)	-3.36	9	0.01^*^
HADS D (0–21) ^↓^	0.00 (2.94)	0.00	6	1.00	-1.50 (2.59)	-1.83	9	0.10
LiSat 11.1 (1–6) ^↑^	5.71 (1.27)	1.18	6	0.28	0.56 (0.73)^a^	2.29	8	0.05^*^
EQ-5D-3L (0–1) ^↑^	0.31 (0.27)	3.03	6	0.02^*^	0.18 (0.36)	1.57	9	0.15
EQ-VAS (0–100) ^↑^	22.14 (5.67)	10.33	6	0.00^*^	6.80(18.83)	1.14	9	0.28
SOC-13 (13–91) ^↑^	2.57 (8.28)	0.82	6	0.44	-1.50 (7.37)^b^	-0.58	7	0.58
WHODAS 2.0 (0–60) ^↓1^	-5.75 (14.82)^c^	-1.42	2	0.29	-1.22 (6.55)^a^	-0.56	8	0.59
COOP/WONKA (0–25) ^↓1^	-5.00 (6.08)^c^	-0.78	3	0.49	-0.88 (5.54)^b^	-0.45	7	0.67

*COPM* Canadian Occupational Performance Measure, *FAI* Frenchay Activity Index, *SES* Self-efficacy Scale, *RNLI* Re-integration to normal living index, *HADS* Hospital Anxiety and Depression Scale, Anxiety (A), Depression (D); Life Satisfaction Scale 11 (LiSat-11); *EQ-VAS* (EQ-Visual Analogue Scale), *SOC- 13* Sense of Coherence 13, *WHODAS 2.0* WHO Disability Assessment Schedule 2.0, The Darthmouth Functional Health Assessment Chart/WONKA (COOP/WONKA)

^↑^ a higher score indicates a better outcome, ↓ a lower score indicates a better outcome

^1^WHODAS 2.0 and COOP/WONKA were measured at the 1.^st^ follow up, not at baseline

^*^Significant level at *p* < 0.05

Missing value: number of missing participants indicated with ^a^ = 1 person, ^b^ = 2 persons, ^c^ = 3–5 persons

**Table 4:** Clinical changes and cut-offs from baseline to the ten-week follow-up within each group

Measures	IG				CG			
	Baseline Mean (SD)	2^nd^ follow-up Mean (SD)	Mean difference	**Clinical change**/ cut of	Baseline Mean (SD)	2^nd^ follow up Mean (SD)	Mean difference	**Clinical change**/ cut of
COPM Performance (1–10) ^↑^	4.22 (1.31)	7.59 (1.55)	**3.37**	** > 2**	2.55 (1.73)	5.17 (3.24)	**2.62**	** > 2**
COPM Satisfaction (1–10) ^↑^	3.47 (1.96)	7.87 (1.86)	**4.40**	** > 2**	2.80 (1.61)	5.76 (3.20)	**2.96**	** > 2**
Barthel Index (0–100) ^↑^	54.29 (26.99)	82.14 (6.36)	27.85	> 95	71 (24.12)	82.50 (24.30)	11.50	> 95
KATZ (0–10) ^↑^	2.86 (1.95)	5.29 (1.70)	2.43	10	4.60 (1.89)	6 (2.75)	1.40	10
FAI (0–45) ^↑^	15.14 (10.96)	8.00 (3.51)	- 7.14	-	20.20 (13.25)	16.70 (9.21)	-3.50	-
SES (18–180) ^↑^	76.86 (28.03)	**105.33 (23.10)**	28.47	**> + 90**	**99.40 (31.83)**	**129.50 (45.29)**	30.10	** > + 90**
RNLI (11–44) ^↑^	29.43 (3.50)	33.86 (4.67)	4.43	^-^	31.40 (9.50)	32.70 (5.52)	1.30	-
HADS A (0–21) ^↓^	4 (3.51)	4 (3.51)	0	> 7	5.70 (4.08)	4.10 (3.87)	-1.60	> 7
HADS D (0–21) ^↓^	3.71 (2.43)	3.71 (3.25)	0	> 7	4.80 (3.49)	3.30 (2.58)	- 1.50	> 7
LiSat 11.1 (1–6) ^↑^	3.71 (1.70)	**4.29 (1.11)**	0.58	**4–5**	4.11 (0.60)	**undefined 4.60 (0.97)**	0.49	4–5
EQ-5D-3L (0–1) ^↑^	0.13 (0.27)	0.43 (0.26)	**0.30**	** > 0.1**	0.36 (0.41)	0.54 (0.34)	**0.18**	** > 0.1**
EQ-VAS (0–100) ^↑^	40 (11.54)	62.14 (10.75)	**22.14**	** > 10**	54.70 (21.41)	61.50 (24.83)	6.80	> 10
SOC-13 (13–91) ^↑^	**64.86 (9.72)**	**67.43 (7.37)**	2.57	**> 61**	**71.50 (5.75)**	**66.30 (12.05)**	-5.20	**> 61**
WHODAS 2.0 (0–60) ^↓1^	38.29 (8.40)	34 (12.94)	-4.29	^-^	31.50 (10.45)	31.25 (11.84)	-0.25	-
COOP/WONKA (0–25) ^↓1^	19.80 (3.27)	15 (4)	-4.80	-	17.20 (5.02)	15.78 (7.78)	-1.42	-

Canadian Occupational Performance Measure (COPM); Frenchay Activity Index (FAI); Self-efficacy Scale (SES); Re-integration to normal living index (RNLI); Hospital Anxiety and Depression Scale (HADS)—Anxiety (A), Depression (D); Life Satisfaction Scale 11 (LiSat-11); EQ-VAS (EQ-Visual Analogue Scale); Sense of Coherence 13 (SOC- 13); WHO Disability Assessment Schedule 2.0 – 12 (WHODAS 2.0); The Darthmouth Functional Health Assessment Chart/WONKA (COOP/WONKA)

Clinically significant level or cut-off score for each instrument: COPM – an increase of 2 is considered a clinically significant change; Barthel and KATZ – higher score = more independence, total independent if scoring + 95 or 10; FAI – Higher score = higher independency, no cut-off score; SES – responses above 5 (1–10) implies confident in performing activities, 18 activities give a mean score of 90; RNLI – higher scores = the better the client perceives integration, no cut-off score. HADS A/D—scores below 7 indicates no anxiety/depression; LiSat – a score of 4–5 = average score, higher score = more satisfied; EQ-5D-3L + VAS – a change more than 0.1 resp. 10 indicates a clinical change; SOC-13 – Swedish average value 61 (SD 9), a higher score equal to a higher sense of coherence; WHODAS 2.0 and COOP/WONKA – lower scores indicates lesser health problems, no cut-off score

^↑^ a higher score indicates a better outcome, ↓ a lower score indicates a better outcome

^1^WHODAS 2.0 and COOP/WONKA were measured at the 1.^st^ follow up, not at baseline

**Table 5:** Comparison between groups regarding mean outcome at baseline and ten-week follow-up, and total mean difference between the groups after ten weeks

Measures	Baseline		*P*-value	After 10 weeks		*P*-value	Mean difference		*P*-value
	IG *n* = 7	CG *n* = 10		IG *n* = 7	CG *n* = 10		IG *n* = 7	CG *n* = 10	
COPM Performance (1–10) ^↑^	4.22	2.55^a^	0.05^*^	7.68	7.25^a^	0.84	3.46	5.00	0.41
COPM Satisfaction (1–10) ^↑^	3.47	2.80^a^	0.46	7.87	5.76^a^	0.15	4.40	3.23	0.46
Barthel Index (0–100) ^↑^	54.29	71	0.20	82.14	82.50	0.97	27.86	11.50	0.20
KATZ (0–10) ^↑^	2.86	4.60	0.09	5.29	6.00	0.55	2.43	1.40	0.37
FAI (0–45) ^↑^	15.14	20.20	0.42	8.00^a^	16.70	0.02^*^	-7.14	-3.50	0.29
SES (0–180) ^↑^	76.86	99.40	0.15	105.33	129.50	0.25	33.50	30.10	0.84
RNLI (11–44) ^↑^	29.43	31.40	0.61	33.86	32.70	0.66	4.43	1.30	0.32
HADS A (0–21) ^↓^	4.00	5.70	0.39	4.00	4.10	0.96	0.00	-1.60	0.12
HADS D (0–21) ^↓^	3.71	4.80	0.49	3.71	3.30	0.77	0.00	-1.50	0.28
LiSat 11.1 (1–6) ^↑^	3.71	4.11^a^	0.57	4.29	4.60	0.54	0.57	0.56	0.98
EQ-5D-3L (0–1) ^↑^	0.13	0.36	0.20	0.43	0.54	0.49	0.31	0.18	0.43
EQ-VAS (0–100) ^↑^	40	54.70	0.12	62.14	61.50	0.95	22.14	6.80	0.03^*^
SOC-13 (13–91) ^↑^	64.86	71.50^b^	0.13	67.43	66.30	0.83	2.57	-1.50	0.33
WHODAS-12 2.0 (0–60) ^↓1^	38.29	31.50	0.18	34.00^c^	31.25^b^	0.72	-5.00	-1.22	0.41
COOP/WONKA (0–25) ^↓1^	19.80^c^	17.20	0.32	15.00^c^	15.78^a^	0.87	-5.75	-0.87	0.40

*COPM* Canadian Occupational Performance Measure, *FAI* Frenchay Activity Index, *SES* Self-efficacy Scale, *RNLI* Re-integration to normal living index, *HADS* Hospital Anxiety and Depression Scale, Anxiety (A), Depression (D); Life Satisfaction Scale 11 (LiSat-11); EQ-VAS (EQ-Visual Analogue Scale); Sense of Coherence 13 (SOC- 13); WHO Disability Assessment Schedule 2.0 (WHODAS 2.0); The Darthmouth Functional Health Assessment Chart/WONKA (COOP/WONKA)

^↑^ a higher score indicates a better outcome, ^↓^ a lower score indicates a better outcome

^1^WHODAS 2.0 and COOP/WONKA were measured at the 1.^st^ follow up, not at baseline

^*^Significant level at *p* < 0.05

Missing value: number of missing participants indicated with ^a^ = 1 person, ^b^ = 2 persons, ^c^ = 3–5 persons

COPM, EQ-5D-3L and EQ-VAS were the only instruments that detected clinically and statistically significant changes both within and between the groups after ten weeks. Several instruments were able to detect significant change at some point within or between the groups, but not consistently throughout the study (Table 3).

COPM and EQ-5D-3L detect a positive clinically significant difference within both groups after 10 weeks, although the IG had a greater increase of the mean score compared to the CG (Table 4). In the COPM performance/satisfaction measures, where a change of two or more points is considered clinically significant [43], 60% in the IG improved their perceived performance and 70% their perceived satisfaction, compared with 55% who improved the performance and satisfaction points in the CG. Both groups improved their EQ-5D-3L score (> 0.1 score), but only the IG improved their EQ-VAS by > 10 units. With EQ-VAS, 100% of the IG increased their score after ten weeks, compared to 50% in CG. In addition, 30% of participants in the CG had reduced their EQ-VAS score during these ten weeks.

When comparing the mean difference between baseline and the ten-week follow up for the two groups, EQ-VAS was the only instrument that could detect a statistically significant change (*p* < 0.03) between the groups, where participants in the IG had a higher increase of their mean score compared to participants in the CG (Table 5).

### Feasibility of ASSIST 1.0

#### Fidelity and adherence

##### Reablement and goal-oriented support

The SDHCS group already considered themselves working according to reablement; they had a person-centred and holistic approach and encouraged the older adults to perform everyday activities to increase their independence. However, prior to ASSIST 1.0, the SDHCS group did not set specific activity goals with the older adults. The SDHCS saw the potential in setting goals and considered it beneficial in their future work. In addition, they felt that the information about what type of support the older adult wanted to have to reach the goals strengthened their collaboration with the older adult.

In the interviews with the older adults from the IG, it was described how the older adults took initiative to work with the goals on their own, hence, without support from the SDHCS, e.g., they conducted the activity on their own, in whole or in part, before the SDHCS arrived. In addition, after the two weeks with the SDHCS, the continuous work with the set goals seemed to be dependent on the motivation of the older adult, their relationship with the regular home care staff, or their contact with family and friends. Older adults who were motivated to improve their performance in the chosen activities worked independently with their goals and did not always need external support. Others needed reminders or support to perform the specific activities; their ability to achieve the set goals was influenced by the collaboration with the staff from the regular home care service or contact with a significant other.

##### Promoting strategies to support the SDHCS

To support the SDHCS in their work of providing ASSIST 1.0 to the older adults, two strategies were incorporated and assessed: workshops and coaching sessions.

The weekly workshops with the SDHCS group were intended to occur once a week at a given time and place throughout the ASSIST 1.0 project. During the first six months (November to May), a total of 18 meetings with the SDHCS were planned. Meetings did not occur during holidays (such as Christmas and Easter). In the end, 12 of 18 meetings were conducted and on ten of these occasions, 100% of the staff were present. Throughout the summer (June to August) no workshops were planned due to vacations, which also affected the workload. During September and October, only three planned sessions were held.

The reasons for cancelling the workshops were often due to a high workload and prolonged time for the visit at the older adult. In addition, information on sick leave, vacations, and other staff-related obstacles was sometimes communicated at a late stage and the session was then either cancelled or conducted with fewer participants.

The content of the workshops included discussions and reflections on the use of reablement in international contexts, how reablement was linked to the existing work provided by the SDHCS group, and how COPM could be used to identify goals based on the older adult´s wishes and condition. However, in the interviews with the SDHCS group only minor fragments of how the new information and knowledge was implemented by the staff were expressed. Although, they found the workshops to be useful. A positive example was that one of the nurse assistants in the SDHCS had started to ask the older adults about their leisure activities and how participation in these activities could be enabled. By doing so, the staff expanded their approach from not only focusing on personal-ADL (P-ADL) or instrumental-ADL (I-ADL) to also include a broader variety of activities in everyday living that were deemed important for the older adult. The staff reflected upon that they did not focus on this life area before, but that they were met with delight from the older adult when doing so.

The coaching sessions were not adhered according to the design of the intervention. This was partly due to the limited time when ASSIST 1.0 was provided by the SDHCS as well as challenges to coordinate the time with the partners involved. Instead, the staff received support and guidance on how to work according to a reablement approach in relation to set goals and requested support during the workshops.

##### QR-codes

The idea of QR-codes as a technological component was generated shortly after the intervention had started; therefore, a smaller delay was anticipated. The QR-code aimed to facilitate the reablement service by enabling easy access to the older adults´ set goals and their preferred support. Although, when the QR-codes were to be distributed in early 2019, security issues on the server caused a breach into the database, and thus, the connection between the QR-codes and unique data points were lost. This issue did not only delay the incorporation of QR-codes by a couple of months, the problems also resulted in that only four participants in the IG was provided with QR-codes. Consequently, the adherence to use QR-codes was not possible to evaluate due to the low number of users and limited amount of data.

##### Context

A political aspect that influenced the fidelity was the financial plan for the SDHCS. The budget was altered at the beginning of the project period, with the consequence that one of the four nurse assistants had to be let go. Furthermore, decisions on changes in the organisation of the SDHCS resulted in new tasks and increased responsibilities; where a considerable higher number of older adults had moderate to severe cognitive impairments or psychological illnesses, as well as more severe physical illnesses. The interviews with the three remaining SDHCS staff members and their manager revealed that these proceedings considerably affected the SDHCS group and their adherence to the ASSIST 1.0 program.

### Acceptability

#### Reablement and goal-oriented support

Data from the interviews and logbooks showed that the SDHCS group was motivated and engaged during their participation in the ASSIST 1.0 program. The ASSIST 1.0 outline was considered possible to align with the SDHCS current work strategy; therefore, they did not have to reorganise their current work, only add a few new components. In addition, reablement was not completely new to the group and they found it easy to relate to the approach and implement it in their current way of working.

Working with goals was perceived as a natural process for the SDHCS staff, even if they did not set specific goals with the older adults prior to the project. Even if they did not set goals, they always asked what kind of support the older adult wished for and needed, as well as what activities the older adult considered to be necessary and important. The staff also strived to ensure that the older adult tried to perform activities in everyday life on their own and not do the activities for them, when possible. Nevertheless, goals identified with COPM raised additional awareness for the staff, e.g., that there are activities beyond the scope of P-ADL and I-ADL as well as how the support to the older adult could be developed based on these goals.

#### Promoting strategies to support the SDHCS

According to the interviews, the SDHCS group’s acceptability of the workshops was mixed. They saw the potential with the workshops as they could gain new and up-to-date knowledge about reablement. Concurrently, they had to prioritize their work with the older adults and manage new work tasks required by the organisation. Hence, this affected their ability to prioritize the workshops.

Since coaching sessions only occurred once for four participants with different staff members, it was deemed difficult to evaluate the acceptability of this supportive strategy.

#### QR-codes

The acceptability of the QR-codes was deemed to be positive. In the interviews and supportive notes from logbooks, the SDHCS group expressed great potential with the QR-codes; how QR-codes could facilitate communication and information with the regular home care providers and how the wishes and needs of the older adults could be highlighted. The SDHCS group also perceived the technology as easy for them to use in everyday practice, that it was discreetly presented in the person’s home, and sufficient to protect the integrity of older adults. Although, one identified barrier was how the information would be transferred to the different home care providers since information transfer from the SDHCS to regular home care was an existing problem.

## Discussion

The result from this feasibility study demonstrates that the ASSIST 1.0 program was generally acceptable in terms of study design, outcome measures, fidelity, adherence, and acceptability; thus, the ASSIST 1.0 program is considered feasible to scale-up and use in a full-scale study as well as implement in a Swedish context. Applying a reablement approach in combination of conducting workshops with setting goals and developing supporting strategies based on these goals as well as provide support to the staff to enhance their implementation of reablement, appears to have been valuable components for the delivery of reablement and to facilitate a personalised support. The ASSIST 1.0 program consists of strategies and methods that has the potential to be implemented and facilitate a structured and organised provision of home care services and also to support the development of a sustainable way of working for home care providers.

Although ASSIST 1.0 was a feasible program, minor revisions and design considerations would be recommended before a full-scale trial is conducted. The following discussion will focus on factors that affected the intervention, and considerations that has to be addressed before scaling-up the program for national/international use, as well as the programs sustainability.

### ASSIST 1.0, a complex intervention

ASSIST 1.0 comprised of multiple components that acted independently and interdependently [39, 47]. It is considered challenging to assess and evaluate potential boundaries between intervention, implementation, and context [47] as they interact with each other on several contextual levels. One example being the SDHCS delivery of the ASSIST 1.0 program, regarding how the intervention components was implemented in the context. This was evaluated on interviews with the SDHCS and their perceived experience of the delivery and the implementation as well as the researchers observations and reflections of different situations to capture contextual aspects such as organisational, ethical and political [46, 47].

In addition, the results should be interpreted with caution as this was a small feasibility study [46]. Even so, the feasibility was conducted to explore uncertainties and optimise the intervention before a larger trial as well as to evaluate how reablement can be adjusted to various constellations and context.

### Context and reablement

Context is an important factor to consider when developing and delivering reablement programs [7, 41, 65]. It is advantageous to create reablement programs that are adjustable to different contexts as the provision of care to older adults differ between contexts, e.g., between countries and between municipalities.

One important aspect to consider in the context is the organisational structure where the reablement program will be delivered. The organisational structure in the specific context of this study altered the intended design [38] in terms of who would provide the ASSIST 1.0 program, as well as what resources were accessible. In the new context, there were aggravating circumstances for involving regular home care providers to deliver the ASSIST 1.0 program, such as potentially working with different home care providers who would be unknown for the research team in advance. This was because the older adults, in this area of Sweden, could choose between > 30 home care providers to assist them at home when they were discharged from the hospital. Hence, it was deemed too complex to enable within the framework of this study. On the other hand, the context enabled the involvement of an SDHCS group. The service from the SDHCS group were provided independently by the municipality and were not affected by who the older adult chose as home care provider after those two weeks.

#### Aligning the reablement approach

Another advantage of having the SDHCS group delivering the ASSIST 1.0 program was that their working method resembled the reablement approach. In this study, initial meetings were held to lay the foundation of a common understanding what reablement entailed. Because of the similarities between the SDHCS groups’ current work method and the reablement concept, the initial meetings were used to align and improve the strategy how the ASSIST 1.0 program would be delivered rather than introducing new working methods that the SDHCS group had to become familiar with. This alignment was considered to be time efficient and enhancing the adherence with and acceptability of the ASSIST 1.0 program.

The workshops provided additional opportunities to deepen the discussions on the concept of reablement with the SDHCS group and further develop their work in accordance with reablement. Hence, it is advised to assess and understand the link between context and implementation to minimize critical gaps between research and practice [47]. Hence, the staffs prior knowledge and pre-understanding of reablement could be essential to explore in advance to tailor the design or supporting constructs when transferring the program to a new context.

### Enhancing the provision of reablement

Two components were considered to support the provision of ASSIST 1.0, setting goals and thereby tailor the provided support and facilitating professional coaching through workshops.

#### Goal-oriented support

Setting goals within different life areas with the older adult seemed to have increased the SDHCS group’s awareness of the extent of activities that could be considered when supporting older adults in activities in everyday life. In addition, using the goals to develop the support in accordance with the older adults wishes and needs, was also considered a useful strategy by the SDHCS group. Both strategies seemed to have enhanced the group’s critical thinking regarding: their current way of providing support, how they could improve their collaboration with the older adult, and how to align their work to the older adult’s wishes and needs [23, 66]. Hence, these outcomes implies a potential need to raise awareness within the social health organisation that activities in everyday living goes beyond the scope of P-ADL and I-ADL. More emphasis might also be placed on what activities the older adult want and need to perform, as well as what type of support that is requested by the older adult, rather than providing a predefined and standardizes support based on general assumptions. Hence, to facilitate the provision of a reablement approach within health and social care, policy documents and national guidelines that governing the work of the home care service should be revised.

#### Facilitate professional coaching

The second component was the facilitation of professional coaching which was conducted via workshops and ‘hands-on’ coaching. These were new supportive strategies that the SDCHS group had to implement into their work method. The SDHCS group considered the workshops to be valuable to acquire new knowledge about reablement, discussing reablement in relation to their current way of working, and receiving guidance on how they could further develop their work with the older adults. However, barriers to conduct the planed workshops were encountered, hence, a third of the sessions were not conducted. One possible explanation may be that the workshops were held during regular working hours and even though the sessions were approved by the manager, the time for these workshops was not facilitated by the organisation. This meant that the staff had to reorganise and revise their work on their own to participate at these sessions. The organisations readiness and capacity was not evaluated in advance but could have influenced the adherence and acceptability of this component [46]. In addition, incorporation of new activities can be problematic in an already strained environment with limited resources [3, 33], where demands and support from the organisation impacts the staffs work situation [67, 68]. These organisational concerns in combination with the organisations readiness and capacity for change [47] could also have influence the delivery, the intervention, and the implementation of the ASSIST 1.0 program. Hence, if possible, it is recommended to evaluate the organisations readiness and capacity for a study or an intervention to identify barriers and adapt the process.

#### QR-codes

In addition to the goals and the workshops, the third component in ASSIST 1.0 was the evaluation of the incorporated QR-codes. Since QR-codes are easy to use, accessible, and inexpensive, the technology was considered likely to be successfully implemented in the context of community-based home care services. However, in this feasibility there were barriers with the start-up and implementation of QR-codes. Although, both the SDHCS group and their manager expressed in the interviews that they considered the QR-codes to be useful, discrete, and respecting the privacy of the older adult. In addition, they saw the potential with using the QR-codes to highlight the older adults wishes and needs. This statement aligns with previous research that has concluded that technological innovations that prioritize the needs of older adult rather than their frailty have been more likely to be accepted by health and social care workers [69]. QR-codes might be a ground-breaking ICT solution to enhance the person-centred care and provide up-to-date information about the older adults wishes and needs. In addition, it might also support the provision of reablement or programs as the ASSIST 1.0. Incorporation of technology could enhance and streamline the delivery of reablement [70], but has until today not been tested. Therefore, a usability study to evaluate the effectiveness, efficiency, and satisfaction of using QR-codes within the context of home care is recommended. In addition, it should be explored how technological innovations, such as the QR-code, can be incorporate in the context of home care.

### Outcome measurements

Regarding the various instruments used in this study, the purpose was to identify useful instrument in relation to the ASSIST 1.0 program and provide guidance to which instruments to include in a larger trial. Although the sample size might influence this outcome, eight out of twelve instruments detected a significant change in either the IG or CG during this relatively short study period.

In previous reablement studies, the most commonly reported outcomes have focused on function abilities [10, 71] rather than on the older adult’s subjective perception of their abilities, self-efficacy, or quality of life. Although, a person’s everyday life is more than just function. The results from this study indicate that instruments that focus on older adult’s subjective perception of their performance, self-efficacy and life quality appears to detect change to a higher degree during a shorter period compared to instruments that focus on function.

In conjunction with setting goals, COPM aligned well with the intent and concept of reablement. COPM provided a strategy to identify activity issues in everyday living which supported the development of realistically and achievable activity-focused goals. In addition, COPM is an evidence-based outcome measure, with sound psychometric properties, that has been used in a diversity of studies for different populations in different contexts [72]. A tools psychometric property and its relation to the population group are important aspects to consider, which has been suggested in previous reablement research [10]. COPM has been used as outcome measure in previous reablement studies, but to a very limited degree [63]. The COPM alignment with the concept and content of reablement could be utilized to guide the structure and the provision of reablement in future studies or implementations.

### Limitations

The recruitment process encountered challenges, mainly cause older adults felt too fatigued to participate immediately after they were discharged from the hospital. A majority wanted to wait a couple of weeks before they could consider participating, as the time in the hospital was perceived as stressful and they wanted to settle in at home before receiving visits. This may imply that it requires efforts by the older adults to adapt to the new situation at home and maybe also an increased support at home after discharge from the hospital.

Due to the small sample size, the results must be interpreted with caution since the outcomes of older adults´ performance, self-efficacy, and well-being are not generalisable. However, the purpose of this study was to identify instruments that were sensitive enough to detect changes over time, not to produce generalizable results. However, a larger sample could result in a more consistent outcome of instruments that could detect a change between and within groups over a shorter period.

Regarding the provision of the ASSIST 1.0 program, there was a limitation that the regular home care services did not receive professional coaching. The reason was mainly because the older adult could choose between > 30 different home care providers who would continue the care after the SDHCS; the research team did not have the resources to coordinate meetings with the selected regular home care providers. Nevertheless, two weeks with additional support and proper guidance may have been sufficient to encourage and strengthen the older adult to continue on their own with the set goals.

Regarding political and organisational barriers, two factors were considered to influence the adherence in ASSIST 1.0. One barrier was an economic change that took place and which created significant concerns for the staff for a couple of months. Due to political decisions, the SDHCS group was reduced from four to three employees. The second barrier was a political and organisational change concerning the SDHCS work tasks and priorities. During the study, the SDHCS wase assigned to more cases of older adults with various cognitive impairments, which was an exclusion criterion in our study. Nevertheless, it could have been considered to include people with cognitive impairments, as this has been done in previous reablement studies [10, 50]. In addition, organisational changes, such as those presented above, are difficult to account for within a complex system, as interdependent actions in various domains will influence the intervention [47].

## Conclusion

The outcome of this study indicates that ASSIST 1.0 is a feasible program to deliver and evaluate where a logical progression would be to conduct a full-scale trial to provide more conclusive evidence regarding the outcomes of ASSIST 1.0 and include an economical evaluation. With minor improvements, the ASSIST 1.0 program has the potential to contribute to the development of home care provision that focus on the older adults wishes and needs and provides a person-centred support that enhances older adults possibility to age in place at home.

To implement reablement in more contexts and enabling greater possibilities for adaptions to the setting, more constellations of how reablement can be delivered has to be assessed and evaluated. This is the first reablement study where a reablement program is delivered entirely by home care staff who receives professional coaching by an OT.

## Data Availability

The datasets generated and/or analysed during the current study are not publicly available given the small number of participants and ensuring their anonymity, but are available from the corresponding author on reasonable request.

## Abbreviations

ADL: Activities in Daily Living
I-ADL: Instrumental Activities in Daily Living
P-ADL: Personal Activities in Daily Living
CG: Control Group
COOP/WONKA: The Darthmouth Functional Health Assessment Chart/WONKA
COPM: Canadian Occupational Performance Measure
EQ-VAS: EQ-Visual Analogue Scale
FAI: Frenchay Activities Index
HADS: Hospital Anxiety and Depression Scale
ICT: Information and Communication Technology
IG: Intervention group
LiSat-11: Life Satisfaction Questionnaire
OT: Occupational Therapist
RCT: Randomized Controlled Trial
RNLI: Re-integration to normal living index
SDHCS: Supported Discharge Home Care Service
SES: Self-efficacy scale
SOC: Sense of Coherence
QR: Quick Response
WHODAS 2.0: WHO Disability Assessment Schedule 2.0
